# Fashionably late: Temporal regulation of HSV-1 late gene transcription

**DOI:** 10.1371/journal.ppat.1010536

**Published:** 2022-06-16

**Authors:** Joseph R. Heath, Jill A. Dembowski

**Affiliations:** Department of Biological Sciences, Duquesne University, Pittsburgh, Pennsylvania, United States of America; University of Arizona, UNITED STATES

## Introduction

Herpes simplex virus type-1 (HSV-1) is an alpha herpesvirus that infects over 60% of the human population [1]. Infection results in a variety of disease manifestations, including cold sores, encephalitis, and keratitis. The HSV-1 genome contains approximately 152 kb of double-stranded DNA and includes over 80 genes [2,3], which are sequentially transcribed by cellular RNA polymerase II (Pol II) [4,5]. Recently, studies using direct RNA sequencing, long-read sequencing, and ribosome profiling have revealed the transcriptional complexity of the HSV-1 genome and demonstrate that the genome actually contains over 200 open reading frames [6–8]. Viral genes are classified into 4 groups depending on their expression kinetics, including immediate early (α), early (β), leaky-late (γ_1_), and true late (γ_2_) genes. HSV-1 DNA replication is a key point in the infectious cycle, as it enables γ_2_ and amplifies γ_1_ transcription [9,10]. Below, we discuss the studies that defined the 4 HSV-1 gene classes and examine how viral DNA replication may facilitate a switch to regulate γ (γ_1_/γ_2_) gene transcription. Although the HSV-1 gene expression cascade was identified over 40 years ago, high-throughput sequencing approaches continue to reveal new insight into how each gene class is regulated [11–15].

### Temporal and replication-dependent expression of HSV-1 genes

**Fig 1:**
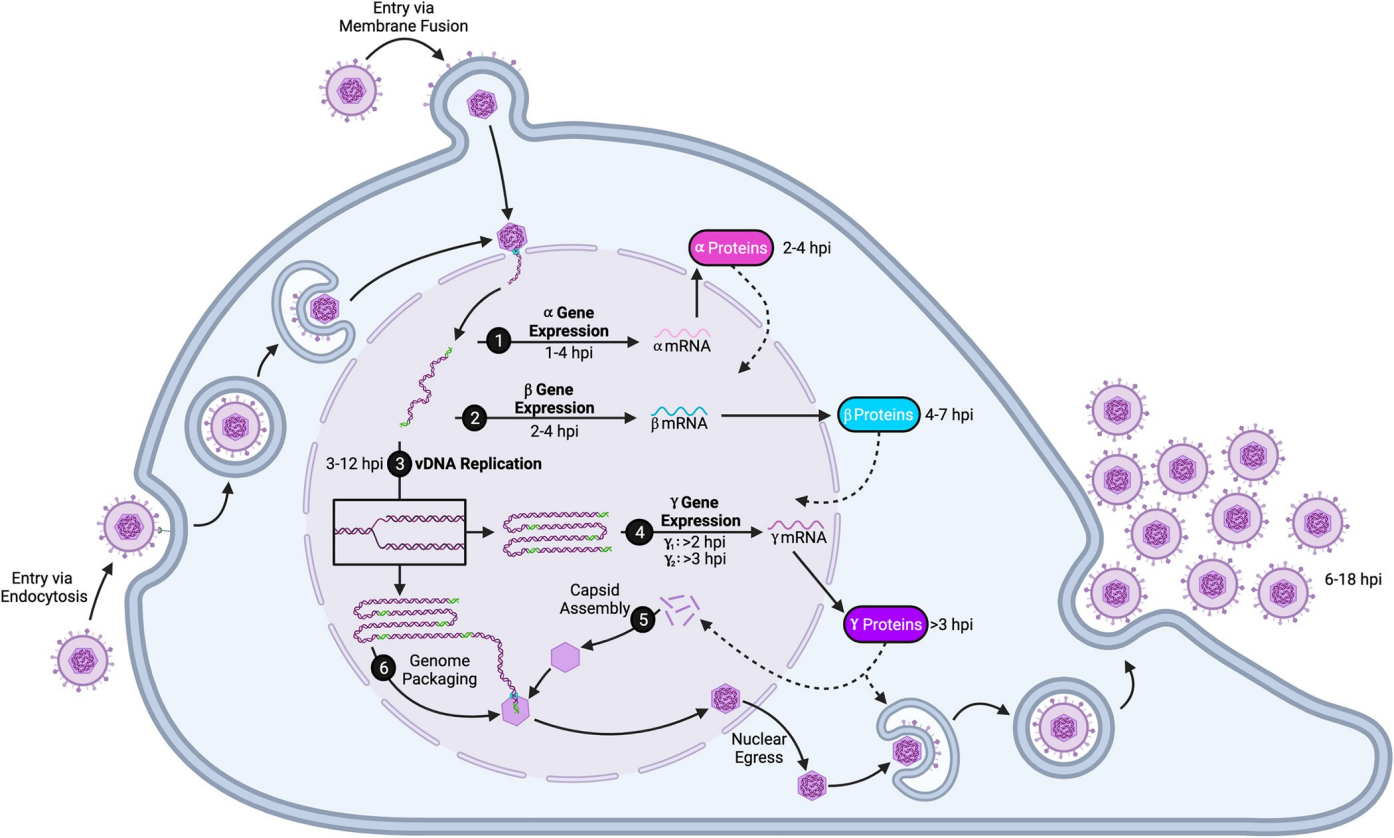
The HSV-1 infectious cycle. Virions enter the cell via fusion with the host-cell membrane or endocytosis. The capsid docks at the nuclear pore and viral DNA enters the nucleus through the portal in the capsid (drawn in teal above). The HSV-1 genome is then expressed: (1) α genes are expressed upon entry; (2) α gene products drive β transcription; (3) after β protein expression, vDNA replication occurs; (4) γ1 gene expression is amplified, and γ2 gene expression is enabled by DNA replication; (5) capsid proteins, which are γ gene products, assemble; and then (6) replicated genomes are packaged into capsids through the portal. Packaged capsids leave the nucleus through nuclear egress and move through the secretory pathway, obtain an envelope spiked with viral glycoproteins, and nascent virions are released from the cell by exocytosis. Transcript and protein timing pictured above are approximated based on previous studies [11,16]. Note that the viral genome forms concatemers during replication as indicated above, and unit length genomes (flanked by green) are packaged into capsids. Also note that some models indicate that the viral genome circularizes after entry into the nucleus. Tegument proteins were omitted for simplicity. Figure created using www.biorender.com. hpi, hours post infection; HSV-1, herpes simplex virus type-1.

HSV-1 genes are temporally expressed during lytic infection. Taking advantage of synchronous infection during high multiplicity infection, the temporal expression of viral genes was previously characterized (**Fig 1**). Early studies demonstrated that α and β polypeptide levels peak between 3 to 4 and 5 to 7 hours post infection (hpi), respectively [16]. γ polypeptides are detected after 3 hpi, and levels continue to increase up to 12 hpi. More recently, RNA sequencing (RNA-seq) was used to measure HSV-1 mRNA levels throughout lytic infection [11]. α mRNAs are detected by 1 hpi and, in general, peak around 4 hpi. β mRNAs are detected by 2 hpi and decline after the onset of viral DNA replication (between 3 to 4 hpi). γ_1_ mRNA is expressed around 2 hpi, and expression is amplified by viral DNA replication. Additionally, γ_2_ mRNA is detected following the onset of DNA replication, with increasing levels observed through 16 hpi. The exact timing of the virus life cycle may vary depending on the cell type.

Further experiments were conducted to define the expression requirements of each gene class. It was found that transcription of α genes does not require de novo viral protein synthesis [16], indicating that cellular proteins and proteins brought into the cell with the infecting virion are sufficient to stimulate α gene expression. On the other hand, both β and γ protein synthesis depend on α protein expression [9,16], including the major viral transcription factor ICP4 [17]. Treatment of infected cells with viral DNA synthesis inhibitors results in the selective inhibition of γ viral gene expression [18–20]. γ_1_ transcripts are expressed in the absence of viral DNA replication and levels increase in a replication-dependent manner [12]. It is possible that this occurs because an increased number of viral genomes within the cell are available to serve as additional templates for transcription. γ_2_ transcription, however, is dependent on viral DNA replication, and viral transcripts are not detectable in the absence of viral DNA replication. This implies that replication results in a switch to enable their transcription. Taken together, these observations indicate that the HSV-1 gene expression cascade is highly coordinated during lytic infection.

### Classification of HSV-1 late genes

RNA-seq was used to globally classify which viral mRNAs are expressed in a replication-dependent manner by comparing expression of viral genes between HSV-1 lab strain KOS and a UL30 (HSV-1 DNA polymerase) mutant that is defective for viral DNA synthesis [12]. Twenty-two γ_1_ mRNAs and 16 γ_2_ mRNAs were identified as either having decreased (γ_1_) or negligible (γ_2_) expression during infection with the UL30 mutant compared to the lab strain (Table 1). These results are consistent with previous reports, reviewed in [21], with the exception that UL42 was more recently classified as a γ_1_, rather than a β gene [12]. In general, α genes encode proteins involved in viral transcription regulation, β genes encode proteins that facilitate viral genome replication, and γ_1_ and γ_2_ genes encode factors involved in virion assembly and exit. Taken together, the regulatory cascade synthesizes viral proteins in an as needed manner during the HSV-1 infectious cycle.

**Table 1:** Classification of leaky late and late genes, along with their general functions [12].

Gene class	Gene name	Protein identity	Function
**Leaky late** (γ_1_)	UL18	VP23	Capsid proteins
	UL19	VP5/ICP5	
	UL35	VP26	
	UL26.5	UL26.5	Capsid scaffold protein
	UL32	UL32	Packaging protein
	UL21	UL21	Tegument proteins
	UL36	UL36	
	UL41	VHS	
	UL46	VP11/12	
	UL48	VP16	
	UL49	VP22	
	US9	US9	
	US10	US10	
	UL27	gB	Membrane glycoproteins
	US4	gG	
	US6	gD	
	US7	gI	
	US8	gE	
	UL45	UL45	Integral membrane protein
	UL11	UL11	Egress proteins
	UL34	UL34	
	UL42	UL42	DNA polymerase processivity factor
**Late** (γ_2_)	UL38	VP19c	Capsid protein
	UL25	UL25	Packaging protein
	UL37	UL37	Capsid assembly proteins
	UL3	UL3	Tegument proteins
	UL16	UL16	
	UL47	VP13/14	
	UL51	UL51	
	US2	US2	
	US11	Vmw21	
	UL1	gL	Membrane glycoproteins
	UL10	gM	
	UL44	gC	
	UL49A	gN	
	US5	gJ	
	UL31	UL31	Egress protein

### Initial rounds of HSV-1 DNA replication are sufficient to license late viral gene expression

An important question is whether ongoing DNA replication is continuously required for γ genes to be expressed. To address this, viral DNA replication was inhibited with acyclovir at 0, 2, 3, 4, or 6 hpi, and the effects on viral gene expression were determined by RNA-seq at 12 hpi [22]. If acyclovir was added before the onset of viral DNA replication (0 or 2 hpi), genome replication did not occur and γ_2_ mRNAs were not expressed. However, if acyclovir was added at 3 or 4 hpi, allowing for 1 to 2 rounds of viral DNA replication as measured by real-time PCR to quantify viral genome number, γ_2_ mRNA levels observed at 12 hpi were similar to an untreated control. These data may indicate that DNA replication is not continuously required for γ_2_ gene expression, and that the alterations that enable their expression occur during initial rounds of replication. It is possible that alterations in viral chromatin or the architecture of the viral genome enable this switch. However, it was recently demonstrated that although chromatin dynamics dictate the transcriptional competency during the onset of lytic infection, it does not appear to play a role in regulating the temporal expression of individual classes of viral genes [13,14].

### Transcription factors associate with nascent viral DNA

Experiments conducted to identify proteins that interact with the HSV-1 genome during viral replication reveal insight into the potential mechanism through which γ gene expression is regulated. Using an adaptation of iPOND (isolation of proteins on nascent DNA), replicating viral DNA was pulse labeled with the nucleoside analog 5-ethynyl-2′-deoxycytidine (EdC) to enable subsequent tagging and purification of replicated viral DNA, followed by the identification of associated proteins by mass spectrometry [22]. In addition to the viral replication machinery, cellular Pol II and transcription regulatory proteins were enriched on EdC-labeled viral replication forks.

iPOND was also used to compare the relative enrichment of individual proteins at viral replication forks with that of nascent viral DNA post replication [22]. For this experiment, an EdC pulse followed by a chase with 2′-deoxycytidine was conducted. This enabled purification of EdC-labeled viral DNA during (pulse) or post (chase) replication. The Mediator complex and the basal transcription factors TFIID and TATA binding protein (TBP) were more enriched on pulse-labeled viral DNA than chased. Pol II and ICP4 were equally enriched on both populations of nascent viral DNA. Additionally, factors involved in cotranscriptional RNA processing were more enriched on chased DNA. The relative enrichment of promoter-binding factors including Mediator, TBP, and TFIID on pulse-labeled DNA; ICP4 and Pol II on pulse-labeled and chased DNA; and RNA processing factors on chased DNA may indicate that the act of DNA replication enables transcription factor binding to promoters, resulting in transcription initiation.

### Late gene promoters and transcription factor binding

**Fig 2:**
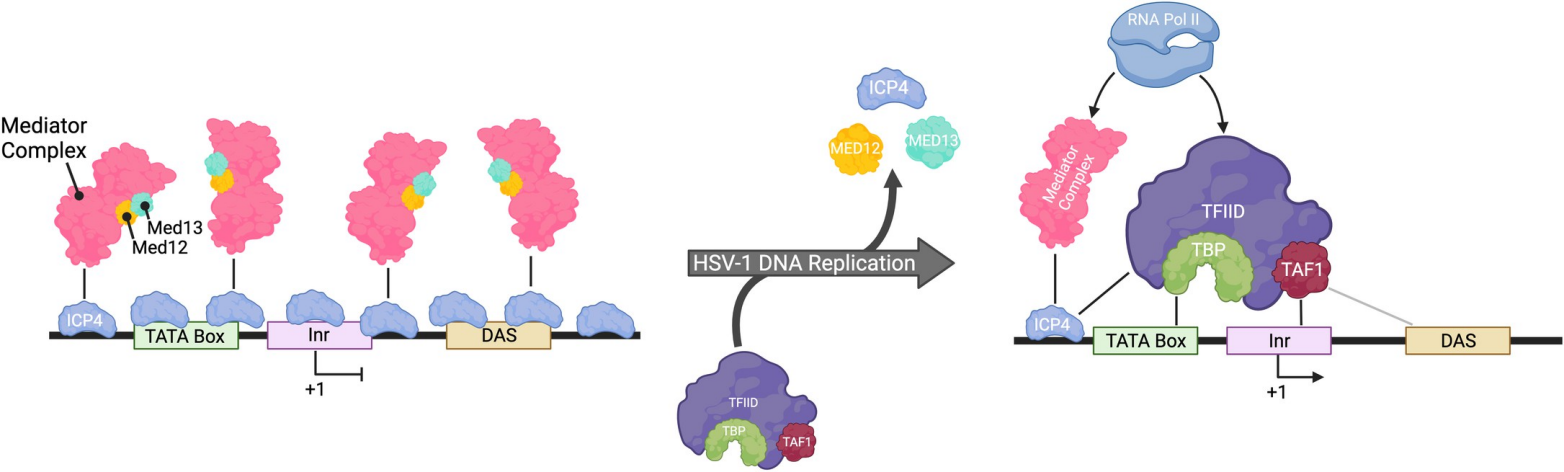
Model of HSV-1 γ_2_ gene promoter interactions before and after DNA replication. HSV-1 γ_2_ promoter regions contain a TATA box, Inr element, and DAS. The TATA box is within the ‒34 to ‒24 region in the promoter depending on the gene. The Inr flanks the +1 site and the DAS is in the 5′ untranslated region. Prior to DNA replication, ICP4 coats γ_2_ genes in a sequence-independent manner. ICP4 recruits the Mediator complex containing Med12 and Med13, which are members of the Mediator kinase domain that can inhibit transcription. Following the onset of DNA replication, ICP4 binding to γ_2_ genes decreases. This may expose the promoter, enabling ICP4 to recruit TFIID containing TBP and TAF1 to the promoter region. It is possible that loss of the Mediator kinase domain following replication licenses γ_2_ transcription. Collectively, Mediator and TFIID may recruit Pol II to γ_2_ genes. Note that this is a proposed model based on current data and other factors likely play a role in this process. Figure created using www.biorender.com. DAS, downstream activation sequence; HSV-1, herpes simplex virus type-1, hpi, hours post infection; Pol II, RNA polymerase II; TBP, TATA binding protein.

Transcription reporters have been used to map key regions of HSV-1 γ_2_ gene promoters that are necessary for replication-dependent transcription (**Fig 2**). The region immediately upstream from the transcription start site and another region downstream in the 5′ untranslated region of the gene are responsible for replication-dependent expression of representative γ_2_ genes [23–26]. This region contains a TATA box, initiator (Inr) element, and downstream activation sequence (DAS), which help to facilitate TBP and TFIID binding to γ_2_ gene promoters in an ICP4-dependent manner [12,13,27–29]. The structure of γ_1_ genes are less well understood, but representative promoters contain SP1 binding sites, a TATA box, and an Inr element [30]. It is likely that the difference between the *cis* elements of γ_1_ and γ_2_ promoters contributes to the differences in their dependence on DNA replication.

To better understand the mechanisms behind HSV-1 gene expression, chromatin immunoprecipitation followed by sequencing (ChIP-seq) was used to map TFIID, TBP, and Pol II binding to viral DNA before and after replication [12]. Acyclovir was also used to investigate transcription factor binding in the absence of viral DNA replication. The binding of TBP and TFIID subunit TAF1, 2 general transcription factors that recruit Pol II to γ_2_ promoters, was enabled by viral DNA replication. TBP binds to the TATA box sequence and TAF1 was enriched near the Inr element of γ_2_ viral genes after the onset of viral DNA replication. In the absence of DNA replication, Pol II was deficient on the promoters and bodies of γ_2_ genes [12,15]. A single genome duplication event was sufficient to recruit TBP, TAF1, and Pol II to γ_2_ genes[12], consistent with previous findings [22]. Additionally, continuous ICP4 expression is required for γ gene expression [13]. These data suggest that DNA replication induces a change to the viral genome architecture, thus increasing the availability of silent γ_2_ gene promoters to general transcription factors resulting in robust transcription.

Using ChIP-Seq, it was found that prior to DNA replication, ICP4 coats the HSV-1 genome at a high density [13]. Following replication, ICP4 is less abundant on the genome, as an increasing number of genomes may compete for the limited amount of ICP4 within the cell. How changes to ICP4 binding to viral DNA before and after the onset of replication facilate γ_2_ gene transcription is not completely understood. One possibility is that ICP4 recruits Mediator to γ_2_ genes before the onset of viral DNA replication in a form that may inhibit transcription (**Fig 2**). In support of this model, Mediator containing the kinase domain has been found to copurify with ICP4 [31]. In addition, in the EdC pulse-chase studies described above, Med12 and Med13 subunits of the Mediator complex associated with replicated viral DNA decreased substantially with increasing time after EdC labeling [22]. These subunits are part of the kinase domain, which blocks Mediator interaction with Pol II to inhibit transcription initiation. Replication may induce a change in the form of Mediator associated with viral DNA enabling a transcriptional switch.

### Remaining questions

The coupling of HSV-1 γ_2_ gene expression to DNA replication is an intriguing concept with several remaining questions. Interestingly, other viruses such as the T4 bacteriophage also couple the expression of late genes to DNA replication [32]. Although unlike HSV-1, continuous DNA replication is necessary. It is currently unknown why initial rounds of DNA replication enable the expression of HSV-1 γ_2_ genes and what specific alterations to the viral genome allow this to occur. As mentioned above, there is likely an alteration to the genome architecture following viral DNA replication that enables a distinct change in the transcriptional competence of the genome. Understaninding the details of this change will further define how γ_2_ gene expression is enabled. It remains to be determined how the abundance of ICP4 or other forms of viral chromatin on the genome contribute to this switch or if other modifications of the genome, such as the repair of nicks and gaps or the formation of recombination intermediates, are involved. While Fig 2 is a proposed model of how γ_2_ genes are regulated, it does not role out the involvement of additional factors or alternate mechanisms. Another interesting observation is that during reactivation from latency, γ_2_ genes can be expressed independent of both prerequisite viral proteins and DNA replication [33,34]. It is unknown what contributes to these differences, but understanding the regulation of HSV-1 gene expression during latency and reactivation could potentially provide insight into factors that contribute to the regulation of γ_2_ genes. While there is significant progress made toward understanding the coupling of HSV-1 γ gene expression with viral DNA replication, there are several remaining paths for future investigation.
